# Prevalence and Factors Associated with Poor Sleep Quality Among Undergraduate Students: A Cross-Sectional Study

**DOI:** 10.3390/ijerph23050585

**Published:** 2026-04-30

**Authors:** Chadayu Udom, Chatkaew Pongmala, Phatcharawadee Srirug

**Affiliations:** 1 Department of Physical Therapy, School of Allied Health Sciences, Walailak University, Nakhon Sri Thammarat 80160, Thailand; 2 Movement Science and Exercise Research Center, Walailak University, Nakhon Sri Thammarat 80160, Thailand; 3 Functional Neuroimaging, Cognitive, and Mobility Laboratory, Department of Radiology, University of Michigan, MI 48106, USA

**Keywords:** sleep quality, musculoskeletal discomfort, young adults, undergraduate student, stress

## Abstract

**Highlights:**

**Public health relevance—How does this work relate to a public health issue?**

- Poor sleep quality is a widespread public health concern among young adults, with this study finding a high prevalence of 65.9% among undergraduate students.
- Sleep quality was associated with modifiable factors, particularly stress and musculoskeletal discomfort.

**Public health significance—Why is this work of significance to public health?**

- Inadequate sleep in emerging adults can lead to long-term detrimental consequences, including impaired mental health and reduced physical well-being.
- The findings emphasize that sleep problems are multifactorial, demonstrating that addressing only behavioral habits may be insufficient without considering psychosocial and somatic triggers.

**Public health implications—What are the key implications or messages for practitioners, policy makers and/or researchers in public health?**

- Student health programs should adopt integrated interventions that combine stress-reduction techniques with ergonomic health strategies to effectively improve sleep quality.
- Sleep health should be prioritized in public health strategies for young adults.

**Abstract:**

Undergraduate students often engage in nighttime activities and electronic device usage that may adversely affect sleep quality and academic performance; therefore, factors associated with sleep quality are important. The objective of this study was to investigate the prevalence and associated factors of poor sleep quality in undergraduate students. Four hundred and five undergraduate students participated in a cross-sectional study and had no history of psychological disorders or use of medications affecting sleep. Data was collected using the Pittsburgh Sleep Quality Index (PSQI), musculoskeletal discomfort questionnaire, electronic device usage questionnaire and Depression Anxiety Stress Scales (DASS-21). Multivariate logistic regression was employed to analyze the factors associated with poor sleep quality. Among the undergraduate students in this study, 65.93% reported having poor sleep quality. The factors associated with poor sleep quality were stress (OR, 1.03; 95% CI, 1.01–1.06) and musculoskeletal discomfort (OR, 1.92; 95% CI, 1.23–2.99) after controlling for other variables. Undergraduate students frequently experience poor sleep quality, with stress and musculoskeletal discomfort being major contributors. These findings highlight the importance of mental health support and stress management programs in improving sleep quality and overall well-being, as well as in preventing long-term detrimental consequences for undergraduate students’ mental health, physical health and academic performance.

## 1. Introduction

Sleep is a basic biological activity that is necessary for human well-being. Previous evidence indicated that poor sleep quality contributed to a wide range of health problems, including fatigue, impaired immune function, and musculoskeletal disorders [1]. Moreover, it is associated with adverse mental health outcomes and an increased risk of neurological conditions such as Parkinson’s and Alzheimer’s [2,3]. It can impair social interactions, problem-solving skills, memory, and concentration, with increasing irritability [4]. Additionally, inadequate sleep may lead to workplace difficulties and a higher risk of traffic accidents due to drowsiness [5]. People with sleep problems are approximately twice as likely to develop cardiovascular diseases compared to those without sleep issues [6], and to develop a significantly higher risk of depression, up to 38 times [7]. Furthermore, there is a strong link between stress and the quality of sleep [8,9]. Previous evidence also suggested a bidirectional relationship between stress and sleep quality, whereby increased stress could impair sleep, while poor sleep quality might further exacerbate stress and emotional dysregulation [10,11].

Poor sleep quality is particularly prevalent among undergraduate students, whose academic, social, and developmental transitions often limit healthy sleep routines. A national survey in the United States reported that university students experienced more than a one-hour reduction in sleep time and a notable rise in sleep problems, often accumulating sleep debt during weekdays and compensating on weekends [12]. The prevalence of poor sleep quality, PSQI scores greater than 5, ranged from 42.4% to 66.3% among undergraduate students [13,14,15] and reached as high as 76% among medical students [8]. In the post-COVID-19 era, PSQI scores greater than 5 were 68.1% [16].

Undergraduate students are typically in the stage of emerging adulthood, a specific population whose sleep quality has not yet been extensively studied. Multiple factors have been associated with poor sleep quality among undergraduate students, encompassing biological, physical, psychosocial, and behavioral factors. Biological factors include gender, changes in hormones [17,18] and circadian rhythm [19]. Physical factors such as medical conditions, musculoskeletal disorders [1], poor physical health and recent illnesses have been shown to negatively influence sleep patterns [14]. Psychosocial factors play a substantial role, including increased irritability, anxiety, tension, high stress levels, depression [13,16,20], strained relationships with family or peers, low social support, limited income, food insecurity, concerns about neighborhood violence, and experiences of discrimination within the university environment [16,20]. In addition, academic-related stressors encompass difficulties during university study and perceived impacts of COVID-19 [16]. Behavioral factors such as smoking habit, alcohol, caffeine and energy drink consumption [21], gambling behaviors, insufficient physical exercise, and excessive electronic device usage have been identified as important contributors [22,23], and transitional factors like being a freshman may also elevate risk due to lifestyle adjustments. The complexity of poor sleep quality in undergraduate students is highlighted by the coupled multidimensional causes, which also point to the need for comprehensive, multifaceted management techniques.

Moreover, in the contemporary digital age, the utilization of electronic devices such as smartphones, tablets, notebooks, and computers has increased significantly, exhibiting no indications of abatement, even during and subsequent to the COVID-19 epidemic. As the world adapted to the ‘new normal,’ prolonged screen time became routine, potentially impacting mental well-being and sleep quality; notably, students enrolled in health science programs were more likely to engage in excessive digital screen use [24]. However, previous studies often focused on medical or health sciences students, leaving other undergraduate groups underrepresented. Less is known about how sleep quality is related to electronic device usage after lights-off and caffeine consumption [22], and multiple domains related to poor sleep quality remain underexplored, especially musculoskeletal discomfort and history of post-COVID-19 infection after the COVID-19 pandemic. The aim of this study was to examine the prevalence of poor sleep quality and its associated biological, physical, psychosocial and behavioral factors among undergraduate students. Electronic device usage may be a factor associated with sleep quality among university students. The findings will be utilized to create evidence-based care, prevention and student counseling programs with the goal of improving sleep quality and promoting undergraduate students’ health.

## 2. Materials and Methods

This cross-sectional study was conducted to investigate the prevalence and associated factors of poor sleep quality among undergraduate students aged 18–23 years. The study was conducted using self-reported questionnaires administered between September and December 2022. The investigation was conducted on Walailak University’s property with approval from the University’s ethics committee (Approval No. WUEC-22-252-01).

### 2.1. Participants

Undergraduate students from Walailak University, Thailand, who expressed interest in participating constituted the study population. The inclusion criteria were both male and female undergraduate students between the ages of 18 and 23 who were able to read and understand the Thai language and were willing to provide informed consent. Individuals with a medical diagnosis or those using medications that could interfere with sleep, such as treatments for Attention Deficit Hyperactivity Disorder (ADHD) and/or depression, were excluded.

The Taro Yamane formula (*n* = N/(1 + N(e)^2^) was used to determine the sample size for this investigation. The total number of Walailak University students was 10,045. The precision error was set at 0.05. Thus, the required sample size was 385. An additional 5% was added to account for incomplete data and potential data collection issues, resulting in a final required sample size of 405. Detailed characteristics of the sample, including gender and age distribution, are presented in Table 1.

### 2.2. Procedures

The first step in this study was to gather four-part self-report questionnaires. Part 1: General Information included gender, age, height, weight, Body Mass Index (BMI), underlying diseases (physical or mental health conditions diagnosed by a healthcare professional), field of study, academic year, COVID-19 infection history, smoking status, the consumption of alcohol, the consumption of caffeine and energy drinks (categorized as none, occasional (<1 time/week), or regular (≥1 time/week) based on frequency [25]), and the musculoskeletal discomfort (during the past 7 days, which was assessed using the Nordic Musculoskeletal Questionnaire). Part 2: Electronic Device Usage included primary device usage (the most used device), types and purpose of electronic device usage, screen time (average total duration of electronic device usage per day) and duration of electronic device usage after lights-off, and applications used. Part 3: The Thai Version of the Pittsburgh Sleep Quality Index (PSQI) consists of 7 domains and 19 questions. A total score of greater than 5 indicates poor sleep quality. This tool assesses the sleep quality over the previous month and demonstrates strong psychometric properties, including a Cronbach’s alpha of 0.84, an intraclass correlation coefficient of 0.89, a sensitivity of 77.78%, and a specificity of 93.33% [26]. Part 4: Depression, Anxiety, and Stress Scale (DASS-21) consists of three subscales, each containing seven items. Each item is rated on a 4-point Likert scale (0–3). Scores for each subscale were summed and multiplied by two according to standard scoring procedures. The final scores were categorized into five severity levels—normal, mild, moderate, severe, and extremely severe—based on established cutoff values. The Cronbach’s alpha value is 0.94 for depression, 0.87 for anxiety, and 0.91 for stress [27].

After the questionnaires were developed, participants were recruited online via Facebook and Instagram, as well as onsite at the university cafeteria using a convenience sampling method. All volunteers were provided with an explanation of the study’s objectives, procedures, benefits and potential hazards before giving written informed consent in accordance with the ethical standard and Declaration of Helsinki. Volunteers were then screened according to the inclusion and exclusion criteria and completed the questionnaires using a form-filling system.

### 2.3. Statistical Analysis

Data completeness was assessed before statistical analysis. The questionnaire dataset contained no missing values, indicating that all participants provided complete responses for all study variables. Therefore, no imputation or case exclusion was required.

Means, standard deviations (SDs), and frequency were used to represent participant characteristics. Univariate logistic regression was conducted, and variables with a *p*-value ≤ 0.2 (anxiety, stress, depression, 1-week prevalence of musculoskeletal discomfort) were chosen for incorporation into the multivariate logistic regression model using an enter method. Interesting factors were also entered into the model including gender, BMI, duration of electronic device usage during lights-off, screen time, alcohol consumption, and caffeine and energy drink consumption. Before performing the binary logistic regression analysis, multicollinearity among the independent variables was assessed in SPSS to ensure that no significant collinearity existed between variables. Multicollinearity was assessed using the variance inflation factor (VIF) and tolerance statistics. All factors had tolerance values greater than 0.2 and most variables had VIFs less than 5. Due to multicollinearity among depression, anxiety, and stress scores (VIF ≥ 5), only stress was retained in the multivariable model based on theoretical relevance and prior evidence.

The odds ratios (ORs) for certain factors were adjusted to account for the impact of every other variable in the model. The 95% confidence intervals (CIs), crude OR, and adjusted OR were computed using binary logistic regression, and *p* < 0.05 was used to determine statistical significance. SPSS Version 29.0.1.0 was used for statistical analysis (Chicago, IL, USA: SPSS Inc.).

## 3. Results

This cross-sectional study aimed to investigate the prevalence and associated factors of poor sleep quality in Thai undergraduate students. The survey included 405 participants.

### 3.1. General Characteristics of the Participants

The results were as follows: 120 participants (29.63%) were males and 285 participants (70.37%) were females. The mean age and BMI were 19.67 ± 1.23 years (range: 18–22) and 22.21 ± 4.61 kg/m^2^ (range: 14.53–45.28), respectively. Most participants were non-smokers (97.53%) and 162 participants (40.00%) were from the health sciences field.

According to the survey on energy drink, alcohol and caffeine consumption, it was found that 350 participants (86.42%) reported no energy drink consumption, while 217 participants (53.58%) and 73 participants (18.02%) reported no alcohol and caffeine consumption, respectively. The primary device used by most participants was a smartphone (80.99%), where the reason for electronic device usage was for educational activities (97.28%) and the average screen time for electronic device usage was 11.31 ± 4.02 h/day. Instagram was the most used application (88.64%). Most participants used electronic devices after lights-off (91.60%) and the average duration of electronic device usage during this period was 37.85 ± 17.64 min/day. In addition, 65.93% of participants had poor sleep quality, as shown in Table 1.

**Table 1:** General characteristics of the participants (*n* = 405).

Characteristics	*n* (%)	Mean ± SD
Age (years)	-	19.67 ± 1.23
BMI (kg/m^2^)	-	22.21 ± 4.61
Gender		
- Male	120 (29.63%)	-
- Female	285 (70.37%)	-
Underlying disease		
- No	370 (91.36%)	-
- Yes	35 (8.64%)	-
Field of study		
- Health Sciences	162 (40.00%)	-
- Others	243 (60.00%)	-
**Lifestyle factors**		
Smoking		
- Non-smoker	395 (97.53%)	-
- Current smoker	10 (2.47%)	-
Alcohol consumption		
- None	217 (53.58%)	-
- Occasional	166 (40.99%)	-
- Regular	22 (5.43%)	-
Caffeine consumption		
- None	73 (18.02%)	-
- Occasional	198 (48.89%)	-
- Regular	134 (33.09%)	-
Energy drink consumption		
- None	350 (86.42%)	-
- Occasional	47 (11.60%)	-
- Regular	8 (1.98%)	-
Electronic device use		
Primary device usage		
- Smartphone	328 (80.99%)	-
- Tablet	306 (75.56%)	-
- Notebook	173 (42.72%)	-
- Computer	20 (4.94%)	-
Purpose for electronic device usage		
- Education	394 (97.28%)	-
- Entertainment	387 (95.56%)	-
- Financial transactions	186 (45.93%)	-
Use of electronic device before sleep		
- No	6 (1.48%)	-
- Yes	399 (98.52%)	-
Screen time (hours/day)		11.31 ± 4.02
Electronic device usage after lights-off		
- No	34 (8.40%)	-
- Yes	371 (91.60%)	-
Duration of electronic device usage after lights-off (minutes/day)		37.85 ± 17.64
Common applications used		
- Facebook	339 (83.70%)	-
- Line	256 (63.21%)	-
- Instagram	359 (88.64%)	-
- Twitter	177 (43.70%)	-
- TikTok	281 (69.38%)	-
- YouTube	289 (71.36%)	-
Health-related outcome		
1-week prevalence of musculoskeletal discomfort		
- No	146 (36.05%)	-
- Yes	259 (63.95%)	-
History of COVID-19 infection		
- No	236 (58.27%)	-
- Yes	169 (41.73%)	-
Sleep quality (PSQI)		
- Good sleep quality	138 (34.07%)	-
- Poor sleep quality	267 (65.93%)	-

### 3.2. Mental Health Issues

A survey on mental health issues using the DASS-21 questionnaire, which assesses stress, anxiety and depression, revealed that most participants experienced normal levels of depression (59.75%) and stress (66.91%), as shown in Table 2. In contrast, anxiety levels were more widely distributed, with only 47.90% classified as normal. Regarding stress, most participants were within the normal range, although mild (11.85%) and moderate (10.37%) levels were also observed.

### 3.3. Factors Associated with Poor Sleep Quality

When multivariable logistic regression was used, the results revealed that stress (adjusted OR: 1.03; 95% CI: 1.01–1.06) and 1-week prevalence of musculoskeletal discomfort (adjusted OR: 1.92; 95% CI: 1.23–2.99) were associated with poor sleep quality in undergraduate students after controlling for other variables, as shown in Table 3.

## 4. Discussion

This study found that 65.93% of undergraduate students had poor sleep quality. Similar to the findings from other studies, poor sleep quality ranged from 42.40% to 76.00% among undergraduate students [8,13,14,16]. The prevalence of poor sleep quality observed in this study was lower than that reported in a previous study conducted in 2017 [8], but higher than that reported in a 2016 study among the same population [13]. These discrepancies may be explained by differences in study populations and timing. The present study included students from multiple academic disciplines, whereas the 2017 study focused on medical students, who tend to experience higher levels of stress. In addition, the current study was conducted following the COVID-19 pandemic, and nearly half of the participants reported a history of COVID-19 infection. Previous evidence suggested that individuals with a history of COVID-19 infection had nearly twice the prevalence of poor sleep quality compared to those without prior infection [15].

Although undergraduate students often engage in nighttime activities and electronic device usage, these factors were not related to poor sleep quality. This may be attributed to the fact that a previous study [23] had primarily examined factors associated with poor sleep quality within a single domain, particularly behavioral factors such as electronic device used, which may have contributed to the observed associations. In contrast, the present study investigated factors across multiple domains simultaneously. Furthermore, previous studies did not use the PSQI to assess sleep quality. In terms of the primary device used by participants, prior studies focused on personal computers, whereas the present study focused on smartphones. In addition, electronic device usage in this study was assessed using self-reported questionnaires, and addiction was not evaluated. Therefore, although participants may have reported a high frequency of electronic device usage, they may not have been addicted, which may explain the lack of an observed association with poor sleep quality.

Among the associated factors entered in the logistic regression model, past week musculoskeletal discomfort and stress emerged as significant factors. Stress levels were positively associated with poor sleep quality. This is consistent with previous studies which reported that stress is well acknowledged as a risk factor for both sleep-related issues and poor health outcomes [28,29,30]. It is a crucial factor in determining systemic health since chronic dysregulation of this system raises the risk of cardiovascular disease, hypertension and endothelial dysfunction [31]. Furthermore, it activates the hypothalamic–pituitary–adrenal (HPA) axis, which causes the hypothalamus to secrete corticotropin-releasing hormone (CRH), the anterior pituitary to release adrenocorticotropic hormone (ACTH), and the adrenal cortex to produce cortisol. By interacting with the autonomic nervous system (ANS), elevated cortisol usually increases sympathetic activity, which raises blood pressure, heart rate and vasoconstriction [2]. Lack of sleep physiologically impairs brain plasticity and emotional control by raising cortisol, upsetting circadian rhythms, and decreasing neurogenesis. Dysregulation of the HPA axis, increased sympathetic activity, changes in melatonin secretion, and changes in pro-inflammatory cytokines including Tumor Necrosis Factor-alpha (TNF-α) and Interleukin-6 (IL-6) are the causes of these effects [2]. Moreover, stress significantly disrupts sleep quality by activating the body’s fight-or-flight response, leading to heightened alertness and hormonal changes like elevated cortisol levels. This often results in longer time to fall asleep, frequent awakenings and reduced deep sleep stages [32,33].

Similarly, participants reporting body discomfort within the past week were almost twice as likely to exhibit poor sleep quality. This finding is consistent with prior evidence that somatic complaints and discomfort are closely related to impaired well-being and sleep disturbances [34]. Poor sleep quality often worsens musculoskeletal discomfort by increasing muscle tension, inflammation, and pain sensitivity, while pain disrupts sleep in a bidirectional cycle. Studies show that people with poor sleep face up to a nine-times-higher risk of symptoms like back or shoulder pain [35,36].

Alcohol, coffee, energy drink intake, smoking and screen time were among the behavioral factors that did not show any significant correlations. While prior research has shown a connection between alcohol and caffeine consumption and poor sleep and health effects [37,38], the lack of significant findings here could suggest that their effects are less pronounced when psychological stress and physical discomfort are considered simultaneously. Similarly, gender and BMI were not significant predictors, although prior research has suggested gender differences and BMI associations with health behaviors [39]. The null findings in this study may reflect differences in sample composition or measurement approaches.

Overall, the findings show that stress and physical discomfort are important factors that are related to poor sleep quality. These findings suggest that strategies for enhancing outcomes in this population may include stress-reduction and physical discomfort-relieving treatments. This study had a few limitations. First, this study utilized a cross-sectional design to examine the prevalence and associated factors of poor sleep quality among undergraduate students, which limits the ability to establish causal relationships. To better elucidate causal linkages and explore potential moderating factors, including sleep hygiene, coping mechanisms and cultural influences, future research should consider adopting longitudinal study designs. Second, participants were recruited using a convenience sampling approach, which may introduce selection bias, as participants who volunteered may differ systematically from non-participants. Random sampling would be preferable, as it ensures equal probability of selection and minimizes selection bias. Third, as data were collected using self-report questionnaires, the results may be subject to self-report bias, including recall bias and inaccurate reporting by participants. Future studies should incorporate objective measurement tools, such as actigraphy or electronic usage logs, to improve data accuracy and reduce reporting bias. Furthermore, examining additional factors, such as smartphone addiction, physical exercise and posture during smartphone use, may provide further insights into factors associated with poor sleep quality.

## 5. Conclusions

Undergraduate students were frequently reported to have poor sleep quality. Stress level and musculoskeletal discomfort were associated factors of poor sleep quality in undergraduate students after controlling for other variables. These findings suggest that addressing stress and musculoskeletal health may be beneficial in supporting sleep health among undergraduate students. However, further longitudinal studies are needed to confirm these causal pathways before definitive clinical protocols can be established.

## Figures and Tables

**Table 2:** Mental health issues: stress, anxiety, and depression from the DASS-21 questionnaire (*n* = 405).

Mental Health Levels	Depression *n* (%)	Anxiety *n* (%)	Stress *n* (%)
Normal	242 (59.75%)	194 (47.90%)	271 (66.91%)
Mild	34 (8.40%)	51 (12.59%)	48 (11.85%)
Moderate	80 (19.75%)	52 (12.84%)	42 (10.37%)
Severe	24 (5.93%)	39 (9.63%)	32 (7.90%)
Extremely Severe	25 (6.17%)	69 (17.04%)	12 (2.96%)

**Table 3:** Factors associated with poor sleep quality (*n* = 405).

Variables	N	Poor Sleep Quality *n* (%)	Crude OR (95% CI)	*p*	Adjusted OR (95% CI)	*p*
Gender						
- Male	120	78 (65.00%)	1.00 (ref)		1.00 (ref)	
- Female	285	189 (66.32%)	1.06 (0.67–1.66)	0.799	1.07 (0.65–1.78)	0.793
BMI	405	-	1.00 (0.96–1.05)	0.990	0.99 (0.95–1.04)	0.799
Screen time	405	-	0.97 (0.93–1.03)	0.303	0.97 (0.92–1.02)	0.254
Duration of electronic device use during lights-off	405	-	1.00 (0.99–1.02)	0.462	1.01 (0.99–1.02)	0.375
Stress	405	-	1.03 (1.01–1.06)	0.008	1.03 (1.01–1.06)	0.010 *
Alcohol consumption						
- None	217	138 (63.59%)	1.00 (ref)	0.519	1.00 (ref)	0.357
- Occasional	116	113 (68.07%)	1.22 (0.79–1.87)	0.361	1.32 (0.83–2.09)	0.237
- Regular	22	16 (72.73%)	1.53 (0.57–4.06)	0.397	1.77 (0.59–5.27)	0.305
Caffeine consumption						
- None	73	49 (67.12%)	1.00 (ref)	0.487	1.00 (ref)	0.376
- Occasional	198	135 (68.18%)	1.26 (0.69–2.29)	0.459	0.93 (0.51–1.69)	0.816
- Regular	134	83 (61.94%)	1.32 (0.83–2.08)	0.240	0.68 (0.35–1.30)	0.244
Energy drink consumption						
- None	352	227 (64.86%)	1.00 (ref)	0.422	1.00 (ref)	0.487
- Occasional	45	35 (74.47%)	1.11 (0.26–4.71)	0.890	1.55 (0.74–3.25)	0.248
- Regular	8	5 (62.50%)	1.75 (0.36–8.45)	0.486	0.85 (0.17–4.25)	0.840
1-week prevalence of musculoskeletal discomfort						
- No	146	83 (56.85%)	1.00 (ref)		1.00 (ref)	0.004 *
- Yes	259	184 (71.04%)	1.86 (1.22–2.84)		1.92 (1.23–2.99)	

* *p* < 0.05, significance and adjusted OR and 95% CI from the multivariate analysis.

## Data Availability

The data are not publicly available due to privacy/ethical restrictions but are available from the corresponding author upon reasonable request.

## Abbreviations

The following abbreviations are used in this manuscript:

PSQI	Pittsburgh Sleep Quality Index
DASS	Depression Anxiety Stress Scales
OR	Odds Ratio
CI	Confidence Interval
COVID-19	Coronavirus Disease 2019
HPA	Hypothalamic–Pituitary–Adrenal Axis
ACTH	Adrenocorticotropic Hormone
ANS	Autonomic Nervous System
TNF-α	Tumor Necrosis Factor-alpha
IL-6	Interleukin-6
BMI	Body Mass Index
VIF	Variance Inflation Factor
